# The effect of group cognitive behavior therapy on Chinese patients with anorexia nervosa: an open label trial

**DOI:** 10.1186/s40337-021-00469-7

**Published:** 2021-09-15

**Authors:** Lian Gu, Yunling Zou, Yue Huang, Qiang Liu, Han Chen, Jue Chen

**Affiliations:** 1 Shanghai Mental Health Center, Shanghai Jiao Tong University School of Medicine, 600 South Wanping Road, Shanghai, China; 2 Shanghai Changning Mental Health Center, Shanghai, China; 3 Counseling Services, University of Nevada, Reno, NV USA

**Keywords:** Anorexia nervosa, Group cognitive behavior therapy, Individual outpatient treatment, Open label trial

## Abstract

**Background:**

The high cost of treatment for anorexia nervosa (AN) and lack of trained specialists have resulted in limited accessibility of effective treatment to patients with AN, which is particularly problematic in China. To increase the accessibility of evidence-based treatment and reduce the cost of treatment, this study aimed to explore the feasibility and efficacy of group cognitive behavior therapy (G-CBT) adapted from enhanced cognitive behavior therapy for eating disorders (CBT-E) in Chinese AN patients.

**Method:**

A total of 78 patients with AN were assigned to G-CBT or individual outpatient treatment (IOT) and received three months of treatment for AN in each condition. Measures of eating pathology, depression and anxiety were administrated to both intervention groups at three time points: baseline, one month of treatment, and end of treatment; results were compared between groups and over time.

**Results:**

There were 70 participants included in the final analysis. Both G-CBT and IOT groups showed significant improvement in eating pathology and associated psychopathology (*p*s < .001) over the course of treatment, but no significant difference in symptom improvement was found between the two groups (*p*s > .05). G-CBT resulted in additional significant improvement in ED psychopathology over the last two months of treatment, and its overall therapeutic effect was influenced by baseline weight and early symptom improvement.

**Conclusion:**

Preliminary findings from this open label trial suggest that G-CBT adapted from CBT-E is feasible in an outpatient setting and as effective as IOT in facilitating weight regain and reducing psychopathology in Chinese AN patients with little evidence for the superiority of either intervention.

*Trial registration*: The current study was registered at clinical trials.gov on September 23, 2018 (registration number NCT03684239).

**Plain English summary:**

People with anorexia nervosa (AN) are known to be unmotivated for treatment and prone to relapse. Recovery from AN often needs intensive, long-term treatment from a specialized multidisciplinary team, which is not accessible for most people in China. Given the increasing incidence of AN and lack of eating disorder (ED) specialists in China, it is important to develop short-term cost-effective treatments for AN. In this study, we explored the feasibility and efficacy of group cognitive behavior therapy (G-CBT) adapted from enhanced cognitive behavior therapy (CBT-E) for people with AN from China. We found that G-CBT was as effective as individual outpatient treatment (IOT) typically provided to AN patients at the research site in facilitating weight regain, improving eating behaviors, and reducing ED and other symptoms. We also found that patients receiving G-CBT made more improvements in cognitive symptoms of the ED, which might help maintain treatment gains and prevent relapse in the long run. This potential long-term advantage of G-CBT needs to be verified in long-term follow-up.

## Background

With economic development and globalization in China, the concepts such as "perfect figure" advertised on the media and promoted by consumerism have become increasingly widespread over the past few decades. The incidence of anorexia nervosa (AN) in China has been on the rise in recent years [1, 2]. For example, according to the clinical record of the Shanghai Mental Health Center (SMHC), the number of outpatients with eating disorders (EDs) in the most recent five years is about three times the number in the previous five years [3].

As a disease most commonly seen in young women, AN often has a chronic disease course and serious health consequences, sometimes leading to death in severe cases [4]. The mortality rates of AN in Chinese populations have ranged from 5 to 20%, appearing to be the highest among all psychological disorders [5]. In addition, AN is difficult to treat and easy to relapse because of its ego-syntonic nature. A significant proportion of patients with AN can only stop disordered eating behaviors but continue to be mentally preoccupied with food and weight after treatment [6]. Although a number of antidepressants and atypical antipsychotic drugs have been used to treat AN, no sufficient evidence for their efficacy has been found in large-scale clinical trials and the overall effectiveness may have been further reduced by poor medication compliance caused by side effects [7]. In contrast, psychotherapy has gained more support for its efficacy and therefore has been considered to be the most effective treatment for AN and widely used in clinical practice [7]. A variety of psychotherapy approaches have been used to treat AN, including cognitive behavior therapy (CBT), family-based treatment, psychodynamic psychotherapy, supportive psychotherapy, family therapy, etc [8]. Cognitive-behavioral and behavioral interventions have received more support from studies of higher evidence levels compared to others. In particular, enhanced CBT for eating disorders (CBT-E) developed by Fairburn and his colleagues [9] has been considered to be one of the most effective therapies for adult ED patients [10, 11].

Compared to patients with other EDs, individuals with AN usually need more intensive, longer-term therapy provided by clinicians with more experience in EDs. For example, CBT-E for AN takes 40 sessions (twice the sessions of CBT-E for other EDs) and is provided twice a week until the patient is consistently regaining weight [9]. Specialized training is also needed for a therapist to understand the complexity of AN and address different challenges presented in treatment, which takes a lot of time and resources. Thus, the high cost of treatment for AN and lack of trained specialists have resulted in limited accessibility of effective treatment to patients with AN, which is particularly problematic in China given the increased incidence of AN and lack of specialized training opportunities. To increase the accessibility of evidence-based treatment and reduce the cost of treatment, group therapy for EDs has gradually emerged over years [12]. Studies in patients with bulimia nervosa (BN) have demonstrated that group CBT (G-CBT) may achieve the same therapeutic effect as individual CBT with a lower cost per patient [13, 14]. Although AN is often more difficult to treat and study than BN [15–17], researchers have done a lot of studies in psychotherapy for AN [18–26]. The research has indicated that in outpatient settings, CBT-E is both viable and promising for adults [18, 19, 21, 22, 25–27] and adolescents [20, 26] with AN. Encouraging results have also emerged for the effectiveness of inpatient CBT-E, particularly in adolescents [23, 24]. However, there has been very limited evidence for the effectiveness of G-CBT for AN [28, 29].

Given the need for more cost-effective treatment for AN in China and lack of sufficient evidence supporting the effectiveness of G-CBT for AN, this study aimed to explore the feasibility and efficacy of G-CBT adapted from CBT-E in Chinese patients with AN. It was also hoped to lay a foundation for the further development of a standardized, culturally sensitive G-CBT protocol for AN that can be disseminated to Chinese clinicians. We hypothesized that G-CBT would be at least as effective as individual outpatient treatment (IOT), treatment as usual (TAU) provided at the research site, in helping AN patients regain weight and improve ED and associated symptoms.

## Method

### Participants

#### Participant recruitment

All research participants were recruited from patients seeking assessment and/or treatment of EDs in the outpatient department of the Diagnosis and Therapy Center for Eating Disorders at Shanghai Mental Health Center. The recruitment started in August 2017 and ended in November 2018. A research clinician approached patients diagnosed with AN, assessed their eligibility for participation in the study, and went through the informed consent process with eligible patients and parents of eligible adolescent patients. After the research clinician explained all aspects of the study and answered questions raised, those who agreed or agreed their children to participate in the study signed corresponding informed consent or assent forms.

#### Inclusion and exclusion criteria

The inclusion criteria of the study were as follows: Han Chinese; 14–30 years old; education level of junior high school or above; a diagnosis of AN based on the Diagnostic and Statistical Manual of Mental Disorders, Fifth Edition (DSM-5; American Psychiatric Association [APA], 2013); no previous experience of nutrition counseling, individual therapy, or group therapy for EDs; capacity to understand the nature of the study and give informed consent/assent. The exclusion criteria included active suicidality, medical instability that requires hospitalization, inability to participate effectively in therapy due to learning disability or limited language skills, or a history of taking psychotropic medication in the past three months.

#### Participant assignment

**Fig. 1:**
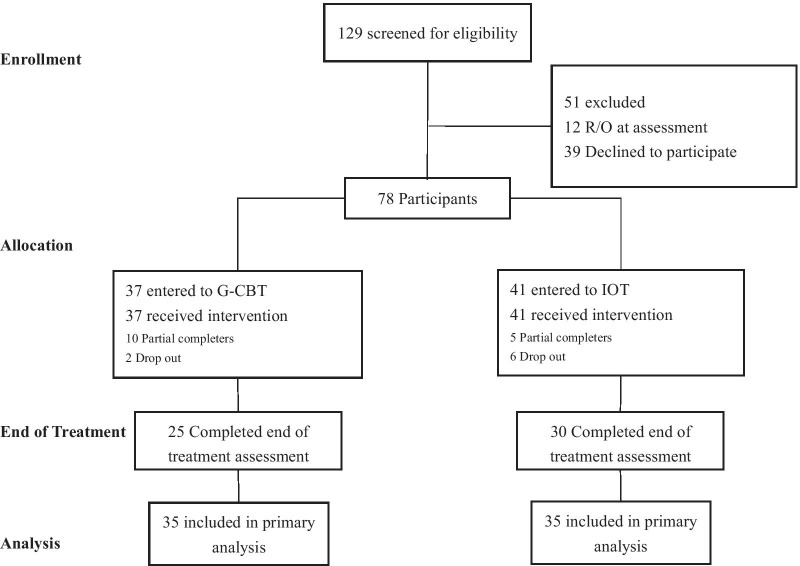
CONSORT diagrams: study flow

A total of 129 patients with AN were recruited and 78 were included in the study. Each participant was assigned a number from the random number table containing an equal proportion of odd and even numbers after entering the study; then participants with even numbers were assigned to G-CBT and those with odd numbers to IOT (Fig. 1).

### Outcome measures

All study participants completed measures of eating pathology, depression and anxiety at baseline, one month of treatment, and the end of treatment, respectively. Their weight and height were also obtained at baseline, after which only weight was measured and shared privately with each participant at the beginning of each individual session or prior to each group session until the end of treatment. The body mass index (BMI) was calculated from measured weight and height and used as one of the outcome measures. Other outcome measures are described below.

#### Eating Disorder Examination Questionnaire 6.0 (EDE-Q 6.0)

The EDE-Q 6.0 is a 28-item questionnaire that assesses cognitive and behavioral symptoms of EDs [9]. It contains four subscales (i.e., restraint, eating concern, weight concern, shape concern) and additional questions asking the frequency of objective binge eating and compensatory behaviors as well as current weight, height, and menstrual status, and use of birth control pills. A global score can be calculated to reflect the overall severity of eating pathology, with a higher score representing more severe eating pathology. The translated measure has demonstrated satisfactory psychometric properties (Cronbach’s α = 0.91) in mainland Chinese patients with EDs [30].

#### Beck Depression Inventory-II (BDI-II)

The BDI-II is a well-established instrument that evaluates the presence and severity of depressive symptoms [31]. It consists of 21 items with each item including four statements scored on a 4-point Likert scale of 0 to 3, (0 indicating no symptom and 3 indicating severe symptom). The total score of the scale is the sum of all item scores and can be categorized into four severity levels: no depression (0–13), mild depression (14–19), moderate depression (20–28), and severe depression (29–63). The Chinese BDI-II has shown good psychometric properties (Cronbach’s α = 0.94) in depressed individuals from mainland China [32].

#### Beck Anxiety Inventory (BAI)

The BAI is a 21-item questionnaire that is widely used to measures common symptoms associated with anxiety [33]. Respondents are asked to rate each item on a 4-point Likert scale of 0 to 3 (0 = “not at all” and 3 = “severe”). All item scores are summed to yield a total score ranging from 0 to 63. The higher the score is, the more severe the anxiety is. The Chinese version of this instrument has been validated in mainland Chinese patients (Cronbach’s α = 0.95) [34].

### Intervention

An initial protocol of G-CBT for AN was developed based on the CBT-E protocol described in *Cognitive Behavior Therapy and Eating Disorders* [9] prior to the study, which was used in all CBT groups of the study. A preliminary investigation conducted before this study showed that long-term psychotherapy is not well accepted in China because some patients live too far away from the research site to stay in Shanghai for long-term treatment and other patients cannot afford the cost of long-term treatment. So we adjusted the original 40-session CBT-E for AN to 10 sessions over 3 months to make it more acceptable to Chinese patients. Small revisions were made to the protocol between the first and second CBT groups to improve its cultural appropriateness and feasibility, such as adding examples of eating behaviors and dietary guidelines applicable for Chinese. The G-CBT for AN used in this study is divided into 3 stages: the initial stage (three weekly sessions with the main focus on establishing therapeutic alliance and enhancing motivation for treatment); the main stage (five weekly sessions with the first three focused on ED behaviors and the last two on ED psychopathology); and the end stage (two sessions every other week to review group content, prevent relapse, and process termination). Each CBT group session lasted 120 min with a 10-min break in the middle and all group sessions were scheduled for weekends over a period of three months.

All CBT groups in the G-CBT condition were co-led by two national registered Chinese psychologists, who received training in CBT-E and developed the protocol of G-CBT for AN prior to the study. A licensed psychologist from the United States who specializes in EDs and is experienced in CBT-E helped design the G-CBT protocol and provided clinical consultation to two group leaders throughout the study. A CBT group was started when the number of participants assigned to G-CBT reached 8 to 12. Prior to the start of each CBT group, one of the group leaders conducted a 60-min interview with each group member individually to build rapport and orient the individual to G-CBT.

In the IOT condition, each participant met with a psychiatrist from the Diagnosis and Therapy Center for Eating Disorders for 10 individual sessions over a three-month period. The first eight sessions occurred once a week in the first two months and the last two every other week for the last month. Each individual session was about 30 min long, involving routine psychiatric care, nutrition consultation, psychoeducation, and supportive therapy [35]. Two patients in the G-CBT group and three in the IOT group were prescribed a selective serotonin reuptake inhibitor (SSRI) during their participation in the study to manage severe depression and anxiety that would otherwise interfere with their effective participation in psychotherapy.

### Data analysis

SPSS 22.0 was used for data analysis in the current study. An intention-to-treat analysis using the last observation carried forward method was performed to impute missing data in incomplete cases who dropped out after having completed the first month of treatment and corresponding assessment. As most data were not normally distributed, medians (*Mdn*) and interquartile ranges (*IQR*) were calculated for variables examined in the study. Friedman tests and Wilcoxon signed-rank tests were conducted to detect within-group changes over three time points. Given the significant differences identified in baseline BMI and depression, Quade analysis of covariance (ANCOVA) was performed to compare symptom changes between G-CBT and IOT groups while controlling for baseline differences. The retention rates of the two intervention groups were compared by chi-square test. To explore potential influence of weight on treatment effect, both G-CBT and IOT groups were further divided into two subgroups (i.e., high weight subgroup and low weight subgroup) based on their median baseline BMI values and Mann–Whitney *U* test was used to compare the pre-post treatment changes in outcome measures between the two subgroups of each intervention group. In addition, a generalized linear model was employed to examine whether early changes in outcome measures (i.e., BMI, EDE-Q 6.0 subscale and global scores, BDI-II score, and BAI score) over the first month of treatment can predict overall change in EDE-Q 6.0 global score at the end of treatment.

## Results

### Participant characteristics and baseline comparison

Among 78 patients enrolled in this study, 37 were randomly assigned to G-CBT and 41 to IOT. As eight patients dropped out of the study before the one-month assessment, only 70 patients (i.e., 35 in the G-CBT group and 35 in the IOT group) were included in the final analysis. The median age of the G-CBT group was 17 years (*14, 19*) and median BMI 16.14 (*14.34, 16.98*). The median age of the IOT group was 16 years (*14, 18*) and median BMI 14.69 (*12.90, 16.53*). There were two male patients in the G-CBT group and none in the IOT group. Baseline comparison between the two intervention groups revealed no significant difference in demographics and symptoms except a significantly higher BMI (*p* = 0.019) and lower BDI-II score (*p* = 0.044) in the G-CBT group than in the IOT group (see Table 1).

**Table 1:** Baseline comparison between G-CBT and IOT groups

Baseline variables	G-CBT group (*n* = 35)	IOT group (*n* = 35)	*χ*^2^/*Z*	*p*
*Gender*				
Male	2	0		
Female	33	35	2.059	.151
Age	17 (14, 19)	16 (14, 18)	− 0.437	.662
Education years	11 (8, 13)	10 (8, 12)	− 0.880	.379
BMI	16.14 (14.34, 16.98)	14.69 (12.90, 16.53)	− 2.343	.019*
EDE-Q 6.0 restraint	1.00 (0.00, 3.20)	1.40 (0.20, 3.20)	− 0.845	.398
EDE-Q 6.0 eating concern	1.00 (0.40, 3.60)	1.20 (0.40, 3.20)	− 0.265	.791
EDE-Q 6.0 shape concern	1.40 (0.60, 2.80)	2.00 (0.80, 2.80)	− 0.742	.458
EDE-Q 6.0 weight concern	1.88 (0.75, 3.88)	2.38 (0.88, 3.88)	− 0.694	.488
EDE-Q 6.0 global	1.79 (0.59, 3.42)	1.84 (0.79, 3.02)	− 0.746	.456
BDI-II	9.00 (2.00, 17.00)	15.00 (11.00, 20.00)	− 2.018	.044*
BAI	7.00 (1.00, 10.00)	5.00 (2.00, 13.00)	− 0.790	.430

G-CBT, group cognitive behavior therapy; IOT, individual outpatient treatment; BMI, body mass index; EDE-Q 6.0, Eating Disorder Questionnaire 6.0; BDI-II, Beck Depression Inventory-II; BAI, Beck Anxiety Inventory

*p < 0.05

### Symptom changes over time within each intervention group

Table 2 presents the outcome measures of both G-CBT and IOT groups at three time points over the course of treatment. Both intervention groups showed significant improvement in ED symptoms from baseline to the end of treatment. Specifically, BMI increased significantly and EDE-Q 6.0 subscale and global scores decreased significantly over the three months of treatment in both groups; at the end of treatment, the median EDE-Q 6.0 global scores of both groups dropped below the clinical cutoff (1.27) reported in a previous study with mainland Chinese ED patients [30].

**Table 2:** Changes in eating pathology and anxiety/depression over the 12 weeks treatment for G-CBT group (n = 35) and IOT group (n = 35), assessed using Friedman tests and Wilcoxon signed-rank test

	Start of treatment	4 Weeks	End of treatment	Friedman tests		
				*χ*^*2*^	*p*	Multiple comparison (Wilcoxon signed-rank test) [*p* < .05]
*BMI*						
G-CBT group	16.14 (14.34,16.98)	16.42 (15.22, 17.57)	17.14 (15.91, 17.96)	39.985	.000	SoT < 4 W < EoT
IOT group	14.69 (12.90, 16.53)	14.87 (13.17, 16.90)	16.44 (14.15, 17.60)	35.717	.000	SoT < 4 W < EoT
*EDE-Q 6.0 restraint*						
G-CBT group	1.00 (0.00, 3.20)	0.20 (0.00, 0.80)	0.00 (0.00, 0.60)	28.727	.000	SoT > 4 W = EoT
IOT group	1.40 (0.20, 3.20)	0.40 (0.00, 1.20)	0.20 (0.00, 1.00)	24.440	.000	SoT > 4 W = EoT
*EDE-Q 6.0 eating concern*						
G-CBT group	1.00 (0.40, 3.60)	0.80 (0.00, 2.00)	0.20 (0.00, 1.00)	29.290	.000	SoT > 4 W > EoT
IOT group	1.20 (0.40, 3.20)	0.40 (0.20, 1.60)	0.60 (0.00, 1.20)	28.358	.000	SoT > 4 W = EoT
*EDE-Q 6.0 shape concern*						
G-CBT group	1.40 (0.60, 2.80)	0.60 (0.00, 1.80)	0.40 (0.00, 1.20)	24.442	.000	SoT > 4 W > EoT
IOT group	2.00 (0.80, 2.80)	1.20 (0.40, 2.00)	1.20 (0.20, 1.80)	13.441	.001	SoT > 4 W = EoT
*EDE-Q 6.0 weight concern*						
G-CBT group	1.88 (0.75, 3.88)	0.88 (0.25, 2.63)	0.63 (0.25, 1.13)	24.434	.000	SoT > 4 W > EoT
IOT group	2.38 (0.88, 3.88)	1.38 (0.50, 2.38)	1.25 (0.25, 2.25)	23.074	.000	SoT > 4 W = EoT
*EDE-Q 6.0 global*						
G-CBT group	1.79 (0.59, 3.42)	0.67 (0.16, 1.61)	0.36 (0.13, 1.00)	21.561	.000	SoT > 4 W > EoT
IOT group	1.84 (0.79, 3.02)	0.81 (0.31, 1.74)	0.66 (0.21, 1.46)	33.132	.000	SoT > 4 W = EoT
*BDI-II*						
G-CBT group	9.00 (2.00, 17.00)	4.00 (1.00, 13.00)	4.00 (0.00, 14.00)	17.770	.000	SoT > 4 W = EoT
IOT group	15.00 (11.00, 20.00)	6.00 (0.00, 16.00)	8.00 (2.00, 12.00)	23.412	.000	SoT > 4 W = EoT
*BAI*						
G-CBT group	7.00 (1.00, 10.00)	2.00 (1.00, 10.00)	2.00 (0.00, 9.00)	7.604	.022	SoT > 4 W = EoT
IOT group	5.00 (2.00, 13.00)	3.00 (1.00, 10.00)	3.00 (0.00, 5.00)	12.365	.002	SoT > 4 W = EoT

G-CBT, group cognitive behavior therapy; IOT, individual outpatient treatment; BMI, body mass index; EDE-Q 6.0, Eating Disorder Questionnaire 6.0; BDI-II, Beck Depression Inventory-II; BAI,eck Anxiety Inventory; SoT, Start of Treatment; EoT, End of Treatment

### Comparison of symptom changes over time between intervention groups

The changes in outcome measures over the first month of treatment and the course of treatment did not differ significantly between the two intervention groups when partialling out baseline differences.

### Influence of baseline BMI on overall symptom changes

As indicated in Table 3, patients in the high weight subgroup (BMI > 16.14) of the G-CBT group demonstrated significantly greater improvement in eating concern, weight concern, and global eating pathology (*p* < 0.05) than the low weight subgroup over the course of treatment. In contrast, there was no significant difference in pre-post treatment changes in outcome measures between the high and low weight subgroups of the IOT group, as shown in Table 4.

**Table 3:** Influence of baseline BMI on overall symptom change from baseline to end of treatment in the G-CBT group

	Low weight group (N = 18)	High weight group (N = 17)	Z	*P* [< .05]
BMI difference	1.22 (0.67, 1.71)	0.77 (0.21, 1.43)	− 1.452	.146
Restraint difference	0.00 (− 2.20, 0.00)	− 0.80 (− 3.20, − 0.20)	− 1.857	.063
Eating concern difference	− 0.10 (− 0.80, 0.00)	− 2.00 (− 3.20, − 1.00)	− 3.674	.000*
Shape concern difference	− 0.50 (− 1.65, 0.00)	− 0.80 (− 3.20, − 0.40)	− 1.709	.087
Weight concern difference	− 1.04 (− 1.94, 0.16)	− 2.20 (− 3.50, − 0.61)	− 2.065	.038*
EDE-Q 6.0 global difference	− 0.20 (− 1.69, 0.06)	− 1.35 (− 3.10, − 0.61)	− 2.527	.012*
BDI difference	− 1.50 (− 8.25, 9.00)	− 7.00 (− 8.00, − 3.50)	− 1.375	.169
BAI difference	0.00 (− 2.25, 3.00)	− 2.00 (− 7.50, 0.00)	− 1.962	.053

G-CBT, group cognitive behavior therapy; IOT, individual outpatient treatment; BMI, body mass index; EDE-Q 6.0, Eating Disorder Questionnaire 6.0; BDI-II, Beck Depression Inventory-II; BAI, Beck Anxiety Inventory

*p < 0.05

**Table 4:** Influence of baseline BMI on overall symptom change from baseline to end of treatment in the IOT group

	Low weight group (N = 18)	High weight group (N = 17)	Z	*P* [< .05]
BMI difference	1.25 (0.46, 1.98)	1.14 (0.21, 2.29)	− 0.099	0.921
Restraint difference	− 0.70 (− 1.65, 0.00)	− 0.60 (− 3.20, − 0.30)	− 1.293	0.196
Eating concern difference	− 0.50 (− 1.60, − 0.20)	− 0.40 (− 2.30, − 0.20)	− 0.050	0.960
Shape concern difference	− 0.50 (− 1.05, 0.20)	− 0.60 (− 1.80, − 0.30)	− 1.177	0.239
Weight concern difference	− 0.81 (− 1.94, − 0.06)	− 0.75 (− 2.75, − 0.13)	− 0.628	0.530
EDE-Q 6.0 global difference	− 0.74 (− 1.31, − 0.17)	− 0.69 (− 2.17, − 0.26)	− 0.908	0.364
BDI difference	− 7.50 (− 14.50, − 3.50)	− 5.00 (− 9.00, 0.50)	− 1.984	0.050
BAI difference	− 2.00 (− 4.50, − 0.50)	− 2.00 (− 7.50, 0.50)	− 0.166	0.868

G-CBT, group cognitive behavior therapy; IOT, individual outpatient treatment; BMI, body mass index; EDE-Q 6.0, Eating Disorder Questionnaire 6.0; BDI-II, Beck Depression Inventory-II; BAI, Beck Anxiety Inventory

*p < 0.05

### Relationship between early changes in treatment and overall treatment outcome

**Fig. 2:**
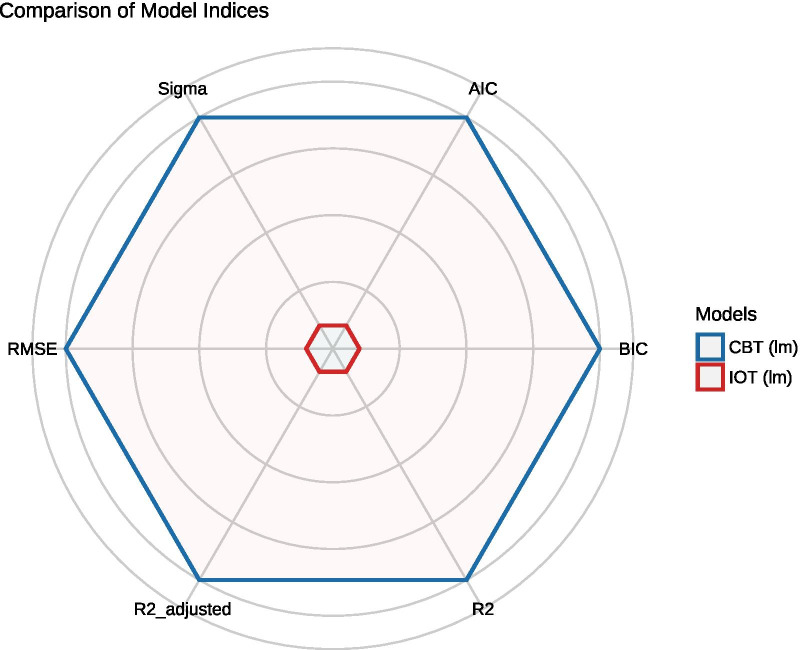
Comparison of model indices between G-CBT group and IOT group

The generalized linear model analysis revealed a larger size overall effect of symptom changes in the first month of treatment on the global eating pathology at the end of treatment in the G-CBT group (*F* = 46.905, *p* = 0.000, adjusted *R*^2^ = 0.904) than in the IOT group (*F* = 4.008, *p* = 0.004, adjusted *R*^2^ = 0.390). The model fit for the G-CBT group is significantly better compared to the IOT group (see Fig. 2). In the G-CBT group, early changes in outcome measures accounted for 90% of variance in global eating pathology at the end of treatment; specifically, improvement in restraint (*t* = 4.245; *p* = 0.000), eating concern (*t* = 5.809; *p* = 0.000), depression (*t* = 2.085; *p* < 0.05) and anxiety (*t* = -3.291; *p* < 0.01) over the first month of treatment each significantly contributed to improvement in global eating pathology at the end of treatment while controlling for other outcome variables.

### Comparison of retention rates between intervention groups

Of the 78 patients enrolled at baseline of the study, 24 (12 in each intervention group) did not complete three months of treatment they were assigned to and 8 of them (2 in the G-CBT group and 6 in the IOT group) dropped out within the first month of treatment, whose data were not included in the final analysis. Among 12 patients who dropped out from G-CBT, 4 were unwilling to continue therapy because they lived too far away from the research site, 5 because they thought the therapy was ineffective, and 3 because they were afraid of gaining too much weight at the end of treatment. Reasons for discontinuing treatment given by 12 patients who dropped out of IOT included not being assigned to G-CBT (2), living too far away from the research site (4), perceiving no symptom improvement (3), and having other unspecified reasons (3). The total retention rate in the study was 69.23%, which was similar to the median retention rate (70%) in ED treatment studies reported previously [36]. Although the retention rate (67.57%) in the G-CBT group was a little lower than that (70.73%) in the IOT group because four more patients were assigned to IOT at baseline, they were not significantly different from each other (χ^2^ = 0.091, *p* = 0.762).

## Discussion

The purpose of this study was to explore the feasibility and efficacy of G-CBT adapted from CBT-E in Chinese patients with AN. No major obstacles were encountered in the implementation of G-CBT during the study with sufficient time to review homework and cover the planned content in each group session, providing preliminary support for the feasibility of G-CBT in an outpatient setting. In line with our hypothesis, both G-CBT and IOT groups showed significant improvement in weight and ED psychopathology (*ps* ≤ 0.001) over the three months of treatment with no significant difference in the degree of improvement between the two groups when controlling for baseline differences (*p* > 0.05). Two patients in the G-CBT group and three in the IOT group were prescribed SSRI during their participation in the study, with no significant difference between the two groups (χ^2^ = 0.215, *p* = 0.643).. It seems that both interventions are equally effective in treating Chinese AN patients. Therefore, G-CBT may be a more feasible, cost-effective option for treatment of AN in China given its relatively low cost per patient and clinician-to-patient ratio.

Significant weight increase was observed in both G-CBT and IOT groups over the first month and last two months of treatment, suggesting no superiority of either in facilitating weight regain. Although both intervention groups achieved significant improvement in ED psychopathology in the first month of treatment, only the G-CBT group demonstrated additional significant improvement in eating, weight and shape concerns as well as overall eating pathology over the last two months of treatment. As ED psychopathology was targeted in the second month of G-CBT, this difference might suggest the effectiveness of G-CBT in reducing ED psychopathology. Because neither intervention specifically addressed ED psychopathology in the first month of treatment, the early improvement in ED psychopathology in both intervention groups might be related to early weight regain, psychoeducation, and therapeutic alliance.

In addition, early symptom improvement over the first month of treatment in the G-CBT group appeared to better predict outcome at the end of treatment compared to the IOT group. Particularly, early improvement in restraint, eating concern, depression, and anxiety were all independently predicted overall improvement in eating pathology when excluding influences from other variables, which was consistent with what was documented in the literature [37, 38]. In contrast, early symptom improvement in none of the examined predictors showed a significant impact on the overall therapeutic effect in the IOT group. It is possible that some other factors in early stage of treatment might have contributed to significant improvement in overall eating pathology at the end of treatment, such as therapeutic alliance [39]. This study also did not find early weight regain as a significant predictor of overall treatment outcome as reported in a previous study [40].

Due to cognitive and personality characteristics of AN patients [16, 41], they are often unwilling to receive treatment themselves. Even if they receive treatment and successfully regain their weight over the course of treatment, they may not make significant improvement in cognitive symptoms and may relapse within a period of time after stopping treatment [42]. CBT has been found to be more effective than general supportive psychotherapy in preventing weight loss after recovery [18], which may be related to its focus on changing the core psychopathology underlying EDs (i.e., overvaluation of shape, weight, eating and their control). In CBT-E, core beliefs and automatic thoughts about food, eating, weight, and shape as well as their importance and relationship are examined and modified, cognitive styles contributing to the development and maintenance of EDs are identified and changed [9], which may help prevent relapse more effectively [26]. Therefore, it is likely that G-CBT will result in greater improvement in ED psychopathology if treatment duration is longer and better prevent relapse if patients are followed up after the end of treatment. This hypothesis seems to be partially supported by additional significant improvement in ED psychopathology achieved by the G-CBT but not IOT group in the last two months of treatment although no conclusion can be drawn due to the lack of post-treatment follow-up in the current study. Future studies should prolong treatment duration and follow up participants after the end of treatment to further examine the proposed hypothesis.

Patients with AN often suffer from starvation syndrome, which can significantly impair cognitive function. When BMI is lower than 15, starvation syndrome is likely to occur and therefore reduce the possibility for AN patients to benefit from CBT. Therefore, some researchers have recommended that the appropriate BMI range for AN patients to receive CBT should be between 15 and 18.5 [43]. In this study, patients in each intervention group were further separated into high and low weight subgroups based on their median baseline BMI. The high weight subgroup of the G-CBT group demonstrated significantly greater improvement in global eating pathology over the three months of treatment than the low weight subgroup, but no significant difference was detected between the high and low weight subgroup of the IOT group. This result appears to provide further support for the finding from previous studies that the weight of AN patients at the time of admission may influence the effect of CBT [21, 44]. Given that low weight does not seem to prevent AN patients from benefiting from IOT in the current study, it may be helpful to regain some weight through IOT before starting G-CBT in severely underweight patients for them to fully benefit from G-CBT.

There were still several limitations in this study. Due to the small sample size and long study enrollment time, this study could not be grouped according to the patient weight and age, resulting in large differences among group members. Thus, there were notable differences between the two treatments (i.e., treatment duration, treatment setting and frequency). We also had to adjust G-CBT to 10 sessions in this study to make it acceptable to Chinese patients, which might have led to insufficient treatment. These factors might have reduced the effectiveness of G-CBT demonstrated in this study and impeded us from drawing broad conclusions, which can be further explored in future studies. It should be noted that a brief version of CBT-E, the ten-session CBT for non-underweight EDs (CBT-T), has been developed and approved to be effective in recent years [45], which may explain the effectiveness of G-CBT in facilitating weight regain and improving ED and associated psychopathology in this study even with the limitations described above.

## Conclusion

In conclusion, G-CBT developed in this study seems to be feasible in an outpatient setting and effectively facilitate weight regain and reduce ED psychopathology in Chinese patients with AN, making it a cost-effective treatment option for this patient population. However, there is limited evidence for the superiority of G-CBT over IOT in treatment efficacy, probably due to the relatively small sample size, short treatment duration, and lack of long-term follow-up in the current study. Future research can further evaluate the long-term effect of G-CBT, determine an appropriate weight to start G-CBT, and identify effective components of IOT that account for its similar effectiveness to G-CBT in treating AN patients.

## Data Availability

The datasets used and/or analysed during the current study are available from the corresponding author on reasonable request.

## Abbreviations

G-CBT: Group cognitive behavior therapy
CBT-E: Enhanced cognitive behavior therapy for eating disorders
AN: Anorexia nervosa
IOT: Individual outpatient treatment
BMI: Body mass index
EDE-Q 6.0: Eating Disorder Questionnaire 6.0
BDI-II: Beck Depression Inventory-II
BAI: Beck Anxiety Inventory
